# Tourist Experiences Recommender System Based on Emotion Recognition with Wearable Data

**DOI:** 10.3390/s21237854

**Published:** 2021-11-25

**Authors:** Luz Santamaria-Granados, Juan Francisco Mendoza-Moreno, Angela Chantre-Astaiza, Mario Munoz-Organero, Gustavo Ramirez-Gonzalez

**Affiliations:** 1 GIDINT, Faculty of Systems Engineering, Universidad Santo Tomás Seccional Tunja, Calle 19, No. 11-64, Tunja 150001, Colombia; juan.mendoza@usantoto.edu.co; 2 SysTémico Research Group, Department of Tourism Sciences, Universidad del Cauca, Calle 5, No. 4-70, Popayán 190002, Colombia; achantre@unicauca.edu.co; 3 GAST, Telematics Engineering Department, Universidad Carlos III de Madrid, Av. de la Universidad, 30, 28911 Madrid, Spain; munozm@it.uc3m.es; 4 GIT, Telematics Department, Universidad del Cauca, Calle 5, No. 4-70, Popayán 190002, Colombia; gramirez@unicauca.edu.co

**Keywords:** CNN, emotion detection, IoT, heart rate, LSTM, recommender system, tourist experience, wearable, xiaomi mi band

## Abstract

The collection of physiological data from people has been facilitated due to the mass use of cheap wearable devices. Although the accuracy is low compared to specialized healthcare devices, these can be widely applied in other contexts. This study proposes the architecture for a tourist experiences recommender system (TERS) based on the user’s emotional states who wear these devices. The issue lies in detecting emotion from Heart Rate (HR) measurements obtained from these wearables. Unlike most state-of-the-art studies, which have elicited emotions in controlled experiments and with high-accuracy sensors, this research’s challenge consisted of emotion recognition (ER) in the daily life context of users based on the gathering of HR data. Furthermore, an objective was to generate the tourist recommendation considering the emotional state of the device wearer. The method used comprises three main phases: The first was the collection of HR measurements and labeling emotions through mobile applications. The second was emotional detection using deep learning algorithms. The final phase was the design and validation of the TERS-ER. In this way, a dataset of HR measurements labeled with emotions was obtained as results. Among the different algorithms tested for ER, the hybrid model of Convolutional Neural Networks (CNN) and Long Short-Term Memory (LSTM) networks had promising results. Moreover, concerning TERS, Collaborative Filtering (CF) using CNN showed better performance.

## 1. Introduction

Internet of Things (IoT) technology enables the integration of wearable and mobile devices to gather historical data from users. Personalized services are designed based on this data to contribute to people’s well-being and quality of life [1,2]. Researchers, in recognition of emotional patterns, find the physiological data of people that is relevant in their daily lives. These devices become a ubiquitous source for providing this data [3]. Emotional detection can be applied in various contexts, including tourism, to improve the tourist experience at destinations [4,5].

On the other hand, tourist expectations from a temporal perspective are analyzed in three phases: before, during, and after the tourist visit [6,7]. This study focuses on the preliminary phase of the visit, which detects the affective condition of people as a contextual factor of a recommender system. To this end, the World Tourism Organization highlights that the tourism industry is more competitive when receptive tourists are more inclined to the emotional benefits than to the physical features and cost of the destination [8].

Preliminarily, the literature review was conducted to identify the components of the emotion-based tourism recommender frameworks [9]. This study showed the gap in integrating physiological data from wearable sensors to detect the affective condition of the user as a relevant contextual factor in the satisfaction of the recommendation. The analyzed approaches mainly considered sentiment analysis techniques to detect emotional states from the social networks reviews. Moreover, these models did not consider low-cost wearables to discover emotional patterns in the user’s daily life.

In this review of the state-of-the-art, it was also found that there is a disparity of formats, emotional states, and physiological signals in the datasets. Wearables of a different range were also used, mainly medium and high-end. Most of these studies took biosignal measurements in controlled experiments [9,10,11,12,13]. As wristbands evolve, they integrate more sensors and with better accuracy. The most common sensors measure heart rate (HR), Galvanic Skin Response (GSR), and temperature. In the context of tourism, the most common wearables are those that are affordable and non-intrusive. Therefore, in this study, the wearable Xiaomi Mi Band was chosen because it is cheap, includes basic physiological sensors, is comfortable, and is easy to use.

One of the research challenges of this study was detecting changes in people’s emotional states in natural and uncontrolled conditions, using wearables with low accuracy in their measurements. To do this, we developed a mobile application to record emotions, independent of the HR record. As a result of this double registration, creating a time series synchronization algorithm called the adjustable and sliding window became necessary.

There are different types of emotions, and therefore their duration and intensity are varied. Norman’s model [14] describes three levels of brain processing to explain the distinct emotional reactions that a person experiences: visceral, behavioral, and reflective. Each person interprets their emotional response according to their identity, culture, personality, and context. Therefore, the automatic detection of emotional states in a time series of physiological measurements became another challenge for this research. In this way, another algorithm was proposed to detect emotional states, known as Emotional Slicing (ES). This algorithm groups HR instances into a time slot to which it assigns an emotional label.

In the previous study of emotional detection on the AMIGOS dataset [15], Convolutional Neural Networks (CNN) [16] were used. Now, in this study, a hybrid Deep Learning (DL) algorithm from CNN and Long Short-Term Memory (LSTM) networks [17,18,19] was implemented for Emotion Recognition (ER) from the ES dataset.

Once the emotion was detected, the Tourist Experience Recommendation System based on the ER (TERS-ER) was developed as the last phase of this study. An interface was designed with the Tourist Traceability Ontology (OntoTouTra) [20] to get the contextual data.

In addition, for the TERS engine, two approaches to Content-Based Filtering (CBF) [21,22] and Collaborative Filtering (CF) based on CNN [2,9,23] were designed to generate the top-N list of Tourist Experiences (TE) recommendations. The TERS engine integrated a user similarity algorithm, selecting candidate users from the ontology based on the profile and contextual data of the wearable user.

This document is organized as follows: Section 2 describes the background to this study and discusses related papers. Section 3 defines the method used for this study. Then Section 4 describes the TERS-ER architecture and its components. Later, Section 5 outlines the TERS-ER performance evaluation and validation. Finally, Section 6 presents the discussion of the results, conclusions, and future work.

## 2. Literature Review

### 2.1. Background

Previously, the performance of some Shallow Machine Learning and Deep Learning algorithms for emotion detection [16], based on the AMIGOS public dataset [15], was compared. In conclusion, it was evidenced that the DCNN architecture showed a better performance in detecting Arousal (0.71 and 0.81) and Valence (0.75 and 0.71) using GSR and Electrocardiogram (ECG) signals (see Table 1). The AMIGOS dataset was collected in tests controlled in the laboratory, using 14 electrodes for the electroencephalogram (EEG), two for the ECG, and one for the GSR. These electrodes were placed on the body of each of the 40 participants. Emotions were elicited through 16 short videos of less than 250 s in length. The resulting dataset is a time series with features corresponding to the physiological signal measurements displayed on 17 channels. Moreover, as labels, it presents the annotations of Arousal, Valence, and dominance [24,25].

Wearables have entered the market in great numbers in recent years [9,26], as have increasingly incorporate sensors that measure physiological signals. The most common sensor present in this type of device is the Photoplethysmogram (PPG) [12,27,28,29], which registers HR signals. The configuration of these devices has aroused scientific interest in detecting the emotional state of a person from these signals [12,13,30]. However, not all wearables have the same level of accuracy in getting the physiological signals [29]. There are devices ranges [10,26]: Expensive devices with high accuracy sensors, principally used for healthcare purposes, for instance, include the Emphatica E4 [11,13,28,31]. Medium range devices with precise sensors have more extensive use, targeted to high-performance athletes, mainly for fitness and sports, and these include, for example, Garmin and Microsoft Band [10,12,32,33]. Affordable devices for all audiences are used for general purposes, for instance, the Xiaomi Mi Band [10,34,35,36,37].

The second stage of the research project [16] corresponds to the study proposed in this paper, applied in the context of tourist recommendations outside the laboratory. The massification of low-cost devices made it possible to reach different contexts, including tourism. Of the three ranges of wearables, the low-cost ones have the highest probability of being used by people who want to have a tourist experience in a destination soon. Then a scientific challenge is created to take advantage of these wearables, which despite their low precision in the measurement of physiological signals, still generate a large amount of data that can be processed with data analytics to discover hidden patterns and trends.

Most of the studies described to the TERS design worked with traditional filtering classifiers such as Collaborative and Content-Based [9]. The second challenge of this study is to integrate the detection of people’s emotional states into the recommender systems. It was traditionally recommended through the reviews of other tourists on lived experiences or based on the context and configuration of the tourist experience in a destination. Nevertheless, this study is intended to refine this type of recommendation according to a person’s emotional state, either to counteract it for negative moods; or to maximize it for positive emotional states. The related work in [9] did not show previous studies regarding a TERS based on the emotional state of a person using HR signals from a low-cost wearable device.

The third challenge of this study is related to how to register the emotional state of a person. In experiments such as the consolidation of the AMIGOS [15] and DEAP [38] datasets, self-annotators and external annotators recorded perceived emotion using a Self-Assessment Manikin (SAM) questionnaire [25]. However, in the daily life context of a person, the researchers of this study developed a mobile application that recorded the mood of the wearable user at different times when he/she felt that an emotion elicitation was being presented. A new challenge arises: synchronizing the time series data of HR measurements with the recording of emotional elicitations. This is achieved by developing a new algorithm, which is the contribution of this study, the sliding and adjustable window algorithm.

### 2.2. Related Works

In this section, ER architectures and methods based on data from wearable devices are analyzed. On the other hand, TERS studies similar to the architecture proposed in this paper are related, based on the previous research of [9].

#### 2.2.1. Affective Detection

Studies have been developed on the detection of the emotional state in different contexts [13,27], most of them in controlled environments and with sensors or specialized wearable devices. The number of participants involved in these types of experiments is around 20 people. Table 2 describes some research for affective detection according to the two-dimensional model of emotions by Arousal (A) and Valence (V) [24]. Also, the specification of the wearable device (physiological sensors and low-cost sensors) and the data collection method (dataset, experiment type, and participants) are given. The last three columns of Table 2 show the physiological signals, the emotion classification approach, and the best performance results of each study.

Meanwhile, Abdel et al. [30] described a method of extracting covariance matrices from EEG signals for the emotion classification using the Log-Euclidean Riemannian Metric (LERM). The study [27] proposed an ER framework by merging multiple physiological signals from the DEAP dataset. Also, they extracted time-domain features from GSR and PPG signals to assess AV detection. These attributes are provided as input to a music recommendation system.

Bulagang et al. [13] used the Empatica E4 device for the collection of HR data from 20 participants, a virtual reality viewer for the elicitation of emotions (emotional quadrants: HVHA, HVLA, LVHA, and LVLA) while the subjects visualized a stream of sixteen 360° videos, for 365 s. Accuracy performance is compared to three methods: Support Vector Machine (SVM), K-Nearest Neighbor (KNN), and Random Forest (RF).

The SAM questionnaire was adopted for the self-assessment of the emotions of 17 participants while watching a series of 24 short videos of affective induction [12]. Inter-Beat-Interval (IBI) features were processed to classify emotional valence using a Bayesian Deep Neural Network (DNN) model.

Deep Learning reduces the complexity of extracting features of traditional statistical techniques because, with manual extraction, the inconvenience of bias in data induction can arise [9]. Another drawback to take into account is the accuracy level of the sensors used. Researchers prefer specialized devices but recognize the need to build new datasets with enough instances, achieved through the use of cheap off-the-shelf wearable devices [12,29].

Unlike the previously described methods for collecting physiological data and emotional labels [9] (see Table 2), this paper proposes data collection methods in people’s daily lives, processing, labeling, and emotional detection based on HR data from Xiaomi Mi Band devices.

#### 2.2.2. Tourist Recommendation Systems

Habitually, Recommender Systems (RS) are becoming more relevant for the decision of the choice of tourist experiences by people [5,6,7,39]. The large Online Travel Agencies (OTAs) incorporate the RS in their systems, and the competitive factor of the agency depends on its effectiveness in the recommendation. Typically RS engines base the prediction primarily on CF and CBF. Considering the maximum number of contextual variables contributes to the accuracy enhancement of the RS [2,4,40].

Preliminarily, in the analysis of the RS literature [9], related works were filtered, whose domain is tourism and that have used sentiment analysis as a contextual factor in the recommendation process (see Table 3). This table incorporates some studies with descriptions of datasets related to tourist destinations (reviews, users, and items), the RS approaches, the similarity metrics (similarity of cosine and Pearson’s coefficient), and the performance evaluation outcomes.

Data from social networks promote emotion analysis and opinion mining from user reviews to determine TE preferences, as well as addressing issues related to a cold start and data scarcity in CF [41,42]. Other studies [41,43] proposed to involve the feelings of the trip as a relevant factor in the experience at the destination using the Term Frequency-Inverse Document Frequency (TF-IDF) technique in the emotion polarity. The studies [42,44] involved the affective, temporal, and location features of users to improve the quality of the RS through a hybrid preference mining algorithm.

Furthermore, in other contexts such as entertainment, business, health care, and smart tourism, the contextual factors of emotions applying sentiment analysis techniques in the classification of reviews have been the subject of research [9]. The user models, based on contextual features extracted from social networks, established the similarity of the users’ preferences of tourist destinations. Also, the application of the algorithms of SVM, KNN, DNN, CNN, and LSTM have been used for the automatic extraction of features and the classification of the mood [9,16].

Unlike the sentiment analysis based on the explicit rating of the reviews in the recommendation processes, this study defines a TERS-ER architecture incorporating the contextual data of the users’ emotions before the tourist visit. For this purpose, a knowledge base of tourist destinations from OntoTouTra [20] is obtained, and the TE are defined according to the AV quadrants. Subsequently, Deep Learning techniques are employed for extracting features and generating the top-N list of TE recommendations.

## 3. Materials and Methods

**Figure 1:**
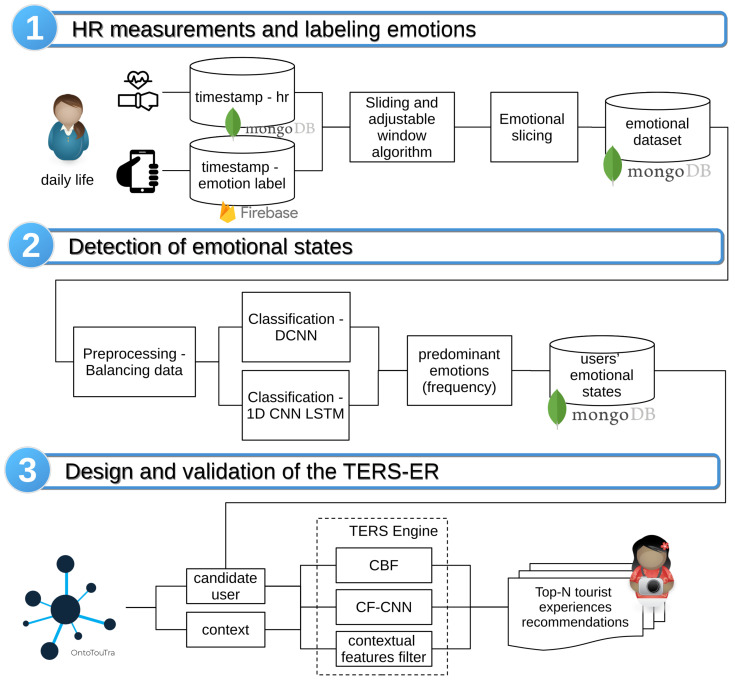
Method overview.

The general process of the methodology used is depicted in Figure 1, which comprises three phases: HR measurements and emotion labeling, detection of emotional states, and TERS-ER design and validation.

### 3.1. HR Measurement and Labeling Emotions

The purpose of this phase was to create an emotional dataset. This dataset is a time series of HR measurements tagged with the emotion felt by the wearable user. The HR register is an objective response to the elicitation of the perceived stimulus in the context, while the emotion register is a subjective response.

As in the similar experiments described in Section 2.2.1, a group of 18 participants was formed, nine men and nine women, whose ages ranged from 18 to 44 years. However, unlike the related work experiments, the study was carried out in contexts outside a laboratory, in the participants’ daily lives. However, three group sessions of controlled elicitation of emotions were programmed to verify the correct recording of the measurements. Each participant was given a Xiaomi Mi Band wristband, and two applications were installed on their mobile device: Master For mi Band (MFB) [45] and MyEmotionBand (MEB). The first app recorded heart rate measurements. The second app was developed in this study to record the person’s emotional states, activities, and location. Before starting the experiment, a group of healthcare professionals assessed the physical and emotional state of the participants. Once the group of participants knew the purpose and procedure of the investigation, they signed the consent for participation. The duration of the experiment was eleven weeks. Short videos were projected for the three group sessions: 19, 19, and 11 videos, respectively. These videos were chosen from the FilmStim repository [46] according to the emotional elicitation of the four AV quadrants [24].

As a result of registering HR measurements, a dataset was created in MongoDB, and from registering the labels, another dataset was created in Firebase. Later, both datasets were synchronized so that the time series coincided with labeling the HR measurements with emotions. For this, Algorithm 1 was developed. Then it was necessary to determine the duration of emotional states [14] using Algorithm 2. Once both algorithms were executed, the emotional dataset was created.

**Algorithm 1.** Sliding and adjustable window for the time series data tagging.
1:
**procedure** getTagDataset
2:    max_size_window=180;3:    **for** *k*, *v* **in** hrData.items() **do**4:        hr={};5:        **for** hrTimestamp, heartRate **in** v.items() **do**6:           $W_{\Delta }=0;$7:           **for** k1, v1 **in** emotion.items() **do**8:               **if** k1 = *k* **then**9:                   **for** key, value **in** v1.items() **do**10:                       **while** $W_{\Delta }$ ≤ max_size_window **do**11:                          window_start = hrTimestamp − $W_{\Delta };$12:                          window_end = hrTimestamp + $W_{\Delta };$13:                          find=False;14:                          **for** eTimestamp **in** v1.keys() **do**15:                              **if** window_start ≤ eTimestamp ≤ window_end **then**16:                                  hr.update(hrTimestamp(v1,hr));17:                                  find=True;18:                                  **break;**19:                              **end if**20:                          **end for**21:                          **if** find = True **then**22:                              **break;**23:                          **else**24:                              hr.update(hrTimestamp(v1,hr));25:                          **end if**26:                          $W_{\Delta }$ = $W_{\Delta }+1;$27:                       **end while**28:                   **end for**29:               **end if**30:           **end for**31:        **end for**32:        hrData.update(hrTimestamp(k,hr));33:    **end for**34:
**end procedure**



**Algorithm 2.** Emotional slicing.
1:
**procedure** buildSlices
2:    timeBetweenInstances=60; sliceSize=30; sliceLimit=20; slicesCounter=0;3:    tagHrList=dataLoad(hrData);previous=tagHrList[0];4:    initSlice();5:    addInstanceToSlice();6:    getMinMaxByImei();7:    **for** row **in** range(1,len(tagHrList) **do**8:        previous=tagHrList[row−1];9:        current=tagHrList[row];10:        **if** previous[0] = current[0] **then**11:           **if** int(current[0]) − int(previous[0]) ≤ timeBetweenInstances **then**12:               **if** $current\left[4\right]{=}^{\prime }movie^{\prime }$ **then**13:                   **if** previous[3] ≠ current[3] **then**14:                       closeSlice();15:                   **end if**16:                   addInstanceToSlice();17:               **else**18:                   addInstanceToSlice();19:               **end if**20:           **else**21:               closeSlice();22:               addInstanceToSlice();23:           **end if**24:        **else**25:           closeSlice();26:           addInstanceToSlice();27:        **end if**28:    **end for**29:
**end procedure**



### 3.2. Detection of Emotional States

**Figure 2:**
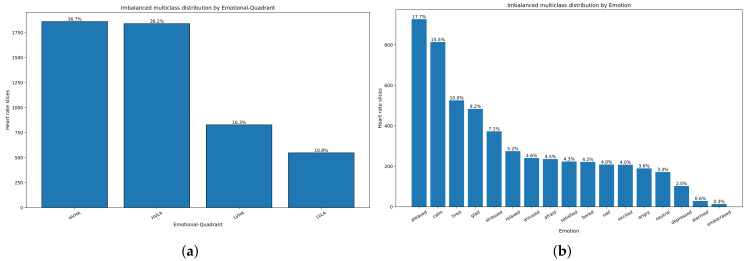
Physiological dataset with a distribution of classes: (**a**) by emotional quadrants; (**b**) by affective states.

In IoT environments, the collection of user datasets can lead to multi-class imbalance, which affects the efficiency and performance of the prediction models. The consolidated dataset in this study presented an unequal distribution in the emotion classes (see Figure 2b) because the participants showed different affective behaviors in their contexts. Likewise, the participants were predisposed to the affective states of pleased, calm, tired, and glad. In contrast to the lower emotional classes of HR records (embarrassed, alarmed, and depressed). In Figure 2a, this same distribution was confirmed for the positive emotion quadrants (HVHA and HVLA) compared to the negative emotion quadrants (LVHA, LVLA).

For this purpose, some studies have used heuristic sampling methods and oversampling techniques for the Multi-class Imbalanced Classification (MIC) using neural networks [47,48,49]. These sampling techniques are based on the nearest neighbor rule of the feature space of each class [49,50]. For the above, the data balancing component sizes the dataset and adjusts the label names by quadrants or emotional states. It also uses class balancing methods to evaluate the performance of affective detection models. That is, the dataset is transformed with the ES instance interpolation methods in the minority classes [51] with the Synthetic Minority Oversampling Technique approaches with K-means (K-SMOTE) [49,52] and TomekLinks (TL) [50]. Subsequently, the combined techniques (K-SMOTE + TL) and oversampling (K-SMOTE) were implemented separately to process the dataset in training [51].

Once the emotional dataset had been balanced, a CNN and LSTM networks hybrid model was used to detect emotion. This model was chosen because it better classified the shallow algorithms’ emotional quadrants (happy, calm, sad, and angry).

An algorithm was designed to determine the predominant emotional state that defined the frequency of the emotion felt by the participants. The results of the execution of this algorithm were stored in a MongoDB collection.

### 3.3. Design and Validation

This phase corresponds to the moment that the wearable device user plans their next TE. The emotional dataset has already been collected, and the predominant emotion of the user has been calculated. So, a TERS is needed that recommends TE according to the person’s emotional state, context, and profile. Therefore, as input sources, the TERS needs the emotional dataset, and concerning the other two requirements, a knowledge base in tourism is used. For this study, OntoTouTra was used.

Initially, similar features were selected among users of the tourist review dataset. To know the profiles of the participants of the experiment, they, in advance, completed a survey with their profile data and TE preferences. With the data from these profiles, similar users were filtered using NLP techniques applied to the username in the ontology to determine its gender. Features of the ontology such as country, ratings, TE, and location were also extracted. The location was compared with the geographic coordinates obtained in the emotional dataset. Then, the similarity was calculated using the Cosine Similarity (CS) metric. In this way, the candidate users were obtained from OntoTouTra.

Two approaches were developed for the TERS engine: A CBF method that determines the similarity between tourist destinations and the other CF-CNN method to relate user preferences. These classification methods processed the filtered information from the destination dataset and extracted the most relevant TE items for the recommendation process. Finally, the list of recommended TE was generated based on the target user’s profile, preferences, context, and emotions.

The following categories of TE [53,54,55] were established:

- Adventure: defines experiences of risky activities such as scuba diving, waterskiing, horse riding, and canoeing.
- Ecological: relates experiences of contact with nature such as hiking, ecological walks, and bicycle tours.
- Entertainment: involves experiences of fun attractions such as movie theaters, theme parks, live music, and sports shows.
- Family: promotes experiences of strengthening relationships between parents and children through attractions on the beach, in the pool, family and children’s games.
- Fitness: promotes wellness experiences and physical activities such as aerobics, gym routine, personal training, and dance.
- Heritage/Culture: promotes experiences of authentic activities such as visits to museums, archaeological sites, and typical food festivals.
- Romantic: involves couples’ romance experiences such as themed dinners, fun in nightclubs and bars.
- Relaxation: involves health care experiences and relaxation activities such as spa, hydrotherapy, sauna, yoga, among others.

The distribution analysis of affective states showed a high rate of participants who registered positive emotions in contrast to negative ones (see Figure 2a). This study assumed that the TE recommendations that people seek are strongly related to increased satisfaction and improving their experiences at destinations [5,6,7]. For this reason, if the detected emotion was negative (sad: LVLA quadrant or anger: LVHA quadrant) or positive (happy: HVHA or calm: HVLA emotional quadrant), the recommender emphasizes positive emotions and mitigates negative ones. For instance, the suggestion for a person who was stressed is the relaxation experience. At the same time, the recommendation for someone calm may be the ecological experience. In this sense, the relationship of emotions with the categories of TE was:

- Happy (HVHA) or sad (LVLA): encourages adventure, family, romantic, and heritage/culture experiences.
- Calm (HVLA) or angry (LVHA): promotes ecological, entertainment, fitness, and relaxation experiences.

## 4. System Architecture

This section describes the operational and structural levels of detail of the TERS-ER architecture. For this purpose, the functional modules, data models, user profiles, and services represented in the context diagrams, containers, components, and classes were identified [56]. Also, according to the IoT architecture [2,19], the TERS-ER layers are:

- The perception layer: It is responsible for collecting HR data using the PPG sensor of the wearable and the emotion and context data from the MEB app installed on smartphones
- The network layer: Transfers HR measurement data using the Bluetooth connection between the wearable and the mobile device. In addition, the smartphone using mobile networks for transferring the emotion and location data of the MyEmotionBand app to the Firebase cloud.
- The service layer: Provides the connections to the Firebase cloud to get the emotion data, the MongoDB server to obtain the HR collections, and the SPARQL endpoint server to retrieve the tourism knowledge base. These datasets are then pre-processed and filtered for the TERS-ER subsystems.
- The application layer: Manages an intelligent RS that displays TE suggestions according to the user’s preferences and contextual factors.

### 4.1. System Context

How satisfying is a particular tourist experience for a person? It depends mainly on the reason for the tourist visit. Often a person looks for options according to information from travel agencies, suggestions from friends, cost of the plans, or the desire to know new destinations. However, the emotional burden that the person manifested before the visit is seldom taken into account. Specifically, to understand the emotional state in a period before the tourist visit, wearable devices are an exciting alternative for capturing physiological and context data. In this way, with the processing of these data, the user’s emotions can be recognized and therefore recommend the appropriate tourist experiences to their affective state. The research question arises: How to design a TERS based on the wearable users’ emotional state in the preliminary visit phase?

**Figure 3:**
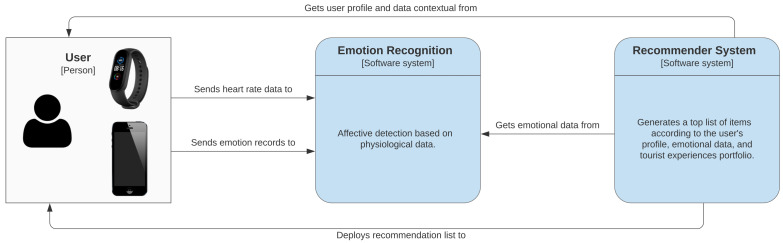
Scenario and context.

Before the visit that can measure in days or weeks, a person in their daily life uses the wristband and mobile devices to record physiological and affective data. In this scenario (see Figure 3), a user, depending on the activity type, (for instance, working, watching movies, resting, traveling, driving, among others) can experience an emotional change caused by various stimuli from the context. Then, through mobile applications, the user can measure HR and record the emotion perceived at that moment (happy, content, sad, calm, angry, and stressed). Afterward, the data from the objective and subjective measurements are processed and analyzed by machine learning (ML) algorithms that detect the person’s affective state.

The recommender system uses the user’s profile (gender and tourist preferences), emotional data, location, and TE portfolio as input items to display destination suggestions. The recommendation list is created from similarity metrics and ML algorithms. Subsequently, the user checks the recommendations of TE according to their emotional state, profile, and preferences.

### 4.2. TERS-ER Architecture

The TERS-ER architecture has two main subsystems. The first is the ER built with the following components: data collection, preprocessing, ES analysis, emotion class balancing, and affective detection using DNN models. The second is TERS, which is implemented with the dataset management components and the recommender engine. This recommender generates the most relevant TE according to the preferences, location, and user emotion in a period before the tourist visit.

**Figure 4:**
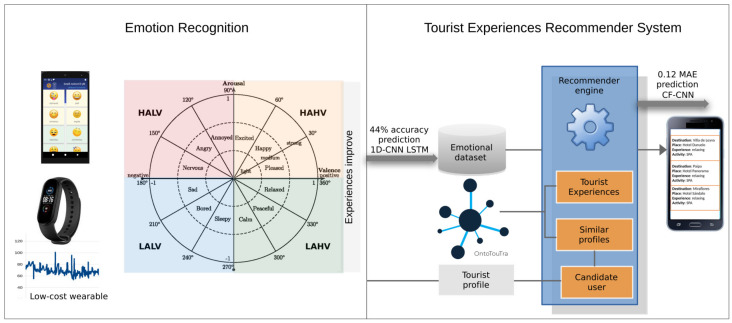
Data model of the TERS-ER architecture.

Figure 4 summarizes the model, and it shows the integration of the subsystems of the TERS-ER architecture.

### 4.3. Technological Container Communication

**Figure 5:**
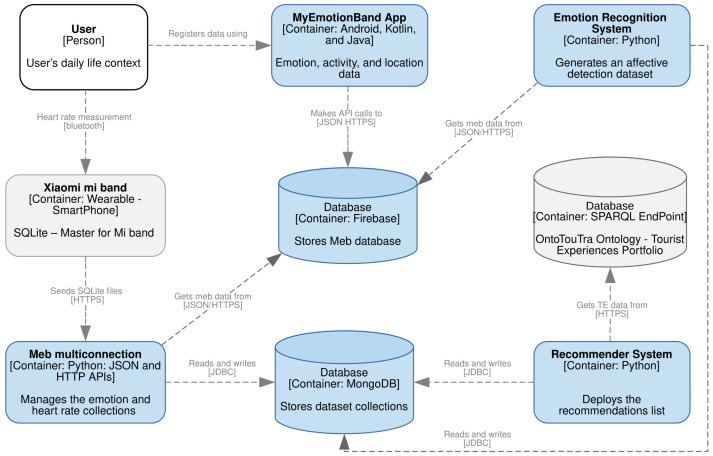
Containers of technological infrastructure.

In this section, Figure 5 depicts the distribution of the technological infrastructure functionalities and the interaction in the TERS-ER subsystems. The following outlines the high-level implementation of the software architecture:

- The users in their context use the Xiaomi Mi Band wristband and the MFB mobile application [45] to store HR data in an SQLite database. In turn, the emotion, activity, and location data is recorded in the MyEmotionBand (MEB) mobile application.
- A real-time database that stores the JSON files of the MEB application in the Firebase cloud.
- An application that manages the connection to the Firebase and MongoDB databases. Also, it handles the collections gathered from wearable and mobile devices.
- A MongoDB database to store collections of HR, emotions, and user profiles.
- An application for ER that generates an affective detection dataset.
- A dataset of the TE portfolio is consulted from the SPARQL endpoint server. This dataset was acquired from the Ontology of Tourist Traceability (OntoTouTra) proposed in [20].
- A recommender engine that processes MongoDB data collections and TE datasets. It then analyzes and displays a list of tourist recommendations.

### 4.4. Apps Architecture

**Figure 6:**
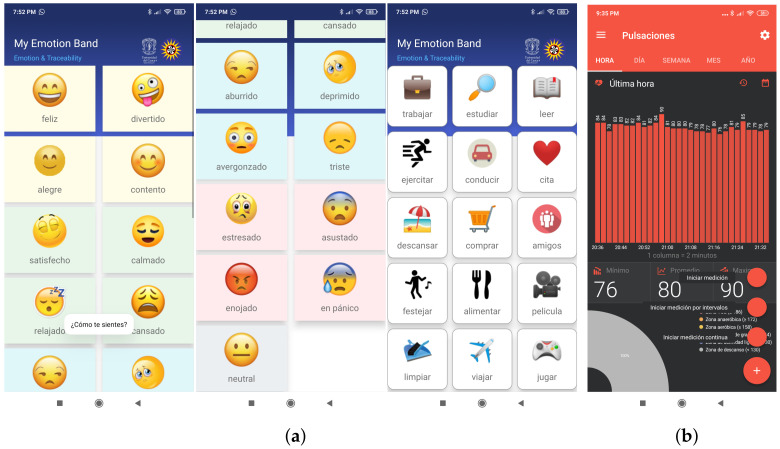
The mobile app’s graphic interface used by the participants: (**a**) MyEmotionBand for recording emotional state, activity, and location; (**b**) MFB [45] for HR measurement.

Figure 6 shows the user interface of the mobile applications developed and used by the participants during the experiment.

The MEB app provided the user interface for recording the affective state (16 emotions and one neutral state) and the activity performed (21 tasks). According to the context of the participants, categorical emotions were associated with activation (Arousal) and emotional polarity (Valence) of Russell’s bi-dimensional model [9,24]. That is, four emotions were defined for each emotional quadrant: happy (HAHV: excited, amused, glad, and pleased), calm (LAHV: satisfied, calm, relaxed, and tired), sad (LALV: bored, depressed, embarrassed, and sad), and anger (HALV: stressed, afraid, angry, and alarmed). The location (latitude and longitude), the date, and time were obtained from the smartphone GPS. In addition, the authentication and synchronization methods were created to store the data on the Firebase server. In particular, the emotional dataset collected 21,000 records from the participants. The SQLite files of the MFB application were converted into CSV files, and a dataset of 1,535,992 HR instances was collected.

In other studies [9,16] the recording of emotions, either by the participant of the experiment or by an observer, was carried out manually on a sheet of paper; this instrument is called SAM [25]. SAM can be used in controlled experiments, but its use is inappropriate in the context of a person’s daily life. For this reason, the MEB app was developed (see Figure 6a). Although the emotion recording is still manual, it is more practical and complete than SAM because the user makes a tap on the emoticon that depicts the emotion that he was feeling at that moment and then another tap on the icon of the activity performed. In this way, MEB correlates the variables of emotion and activity.

### 4.5. Sliding and Adjustable Window Algorithm

**Figure 7:**
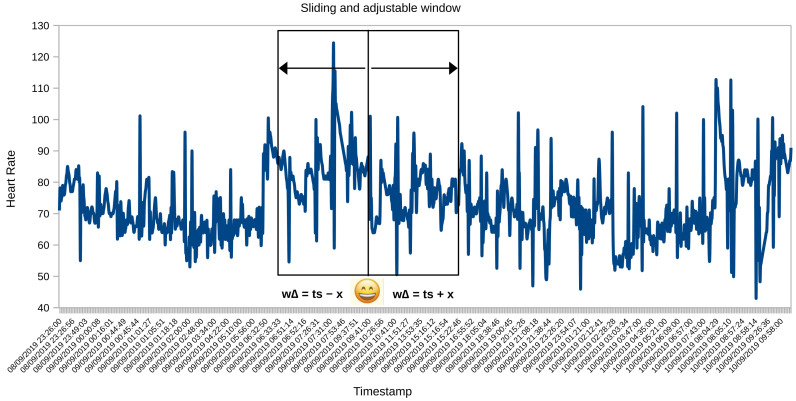
A participant’s sample of HR data with the configuration of a dynamic window adjusted to the HR and emotion timestamp.

The preprocessing of the datasets was made with the synchronization algorithm called a Sliding and Adjustable Window. Because it uses the time series of each participant’s HR and emotional state, this algorithm is sliding because the timestamp window is located in the segment that contains data for both datasets. It is adjustable because the size of the timestamp window is configured depending on the behavior of the data (see Figure 7). The Algorithm 1 loads the two MongoDB data collections (HR and emotion) to tag the emotion in each participant’s HR instances:

- Initially, set up a loop to iteratively go through the HR instances dictionary of the participants.
- Obtains the timestamp and HR measurement of each record. Defines a time variable ($W_{\Delta }$) that controls the window size setting.
- Establishes an iterative cycle through the dictionary of the experiment participants’ emotion, activity, and location.
- Gets the HR and emotional data that correspond to the same participant.
- If the emotion label is not found within the maximum window size (for example, 180 s), it loops through the collection of emotions and gets the tag that matches the timestamp of the HR instance. If the label cannot be found, the size of the window is increased.
- Then, set the emotion tag on HR time series instances. In addition, it adds the activity data and geographical location in the HR dictionary.
- Finally, build a new collection in MongoDB with the HR dataset labeled.

Hence, the method (see Algorithm 1) that we developed is adaptive and dynamic to the time series windows of the physiological and emotional datasets.

As a result of the preprocessing, a tagged data collection of 218,297 records (documents in JSON format) was generated. The data structure is made up of a document identifier (_id), a participant number (IMEI), the emotion timestamp (emotionts), the affective state (emotion), type of activity (activity), HR (hr), location (longitude and latitude) and HR timestamp (hrTimestamp).

### 4.6. Emotional Slicing Algorithm

The size of the segment parameterizes the Emotional Slicing (ES) algorithm (by default 30 HR instances), the time between instances (for example, 60 s), and the limit size of instances (for default 20). The algorithm loads the MongoDB HR collection, consolidates the labeled instance blocks, and generates the physiological dataset used in the affective detection module. This algorithm was created for detecting the duration of the emotion (See Section 3.1).

Algorithm 2 has the following activities:

- Loads the preprocessed collection into a list and gets the first HR instance.
- Initializes a new physiological slice.
- Add the values to the HR, timestamp, and emotion vectors.
- Creates a list with the minimum and maximum HR values for each participant to normalize the data.
- Browses the records of the HR collection. Each iteration verifies that the instance belongs to the same emotional slice of the participant and complies with the limit size of instances. It controls that when the activity is a movie and has the same emotion, it adds the instance to the data vectors. It checks the addition of new instances to other activities that meet the established parameters.
- Then, creates the list of affective segments with the predominant emotion of the HR instances.

Furthermore, the algorithm uses the duplication time-series values technique to adjust the number of HR instances (for instance, a record of 20 HR instances repeats the initial sequence of the vector until it completes the default size of 30). Lastly, the new collection of 5247 ES is stored in MongoDB. The data structure of each participant (JSON format) handles a vector of timestamp and affective segment data (_Id, imei, instances number, slice duration, activity, emotion, longitude, and latitude), together with an HR vector normalized through the linear transformation function depicted in Equation (1) [19]. This method reduces the standard deviation in the data and suppresses the event of outlier values.

(1) $$\begin{array}{c} x_{normalized}=\frac{x-x_{min}}{x_{max}-x_{min}} \end{array}$$

where:

*x*        = measurement of a user’s heart rate;

$x_{min}$ = minimum measurement of all a user’s heart rates;

$x_{max}$ = maximum measurement of all a user’s heart rates;

### 4.7. DNN Models

**Figure 8:**
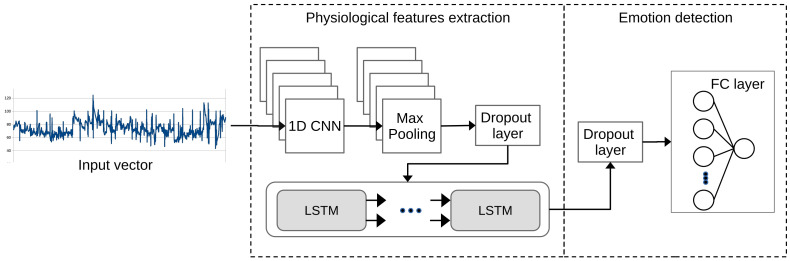
1D CNN LSTM Architecture.

The ER component defines the DNN approaches for detecting affective states based on HR data (see Figure 8) using the Deep Convolutional Neural Network (DCNN) model [16]. This model was built by stacking four 1D CNN layers that reached emotional patterns of physiological signals and three Fully Connected (FC) Layers to predict emotion. Furthermore, two models based on 1D CNN and LSTM architectures [17,19] were defined. Initially, both models used a 1D-CNN to extract the emotion features related to the input vectors. The convolution has a kernel size of 10 and a filter of 128. The second MaxPooling layer reduces the dimensionality of the feature map and has a pool size of two. The first model uses a third flatten layer to convert the feature map into a one-dimensional vector. Then, the fourth FC layer that receives the learned features is connected.

On the other hand, in the second model (Figure 8), after the connections of the 1D CNN and Maxpoling layers, a third dropout layer of 0.5 is added as an exclusion mask to the LSTM that can improve the generalizability. This fourth LSTM layer learns the order of the contextual dependencies of the local features entered. Then, in both models, the 0.5 dropouts fifth layer prevents overfitting during model training and transfers the learned features to the FC sixth layer. Besides, the Rectified Linear Unit (ReLU) activation function is used in the middle layers of the network. While in the output layer, the Softmax trigger function defines the predicted emotion of the multiclass classification.

### 4.8. Recommender Components

**Figure 9:**
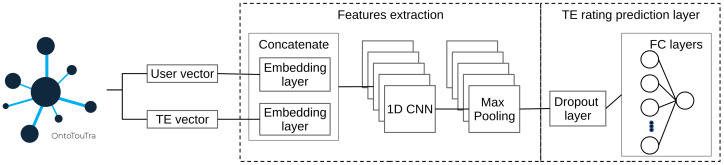
Collaborative Filtering based on 1D CNN and FC layers.

The CBF approach computes the similarity between all the pairs of hotels (see Equation (2)) with the scalar product of categories of TE (binarized vector of TE), location (longitude and latitude), description (summary of services related to TE), and the hotel review tags (for instance exceptional, fantastic and outstanding). The CS metric determines the likeness between TE categories, and the Haversine distance establishes the closeness of locations. On the other hand, the fuzzy match metric [57] compares the description of hotels, and the Python tool “difflib.SequenceMatcher” measures the similarity of the categorical rating of the reviews. As a result, a matrix correlates the similarity of the hotel ratings during the model training and estimates the prediction of the hotel rating for a user. Furthermore, the KNN algorithm derived from the AlgoBase class [21] was used. Afterward, the split of the dataset, the evaluation of the recommendation algorithm’s performance is backed up in the evaluation framework proposed in [22].

(2) $$\begin{array}{cc} TE(h_{i},h_{j}) & =1-spatial.distance.cosine(te\left[h_{i}\right],te\left[h_{j}\right])\\ Location(h_{i},h_{j}) & =mt.exp(-haversine\left(te\left[h_{i}\right],te\left[h_{j}\right]\right)/1.0e3)\\ Category(h_{i},h_{j}) & =SequenceMatcher\left(cat\left[h_{i}\right],cat\left[h_{j}\right]\right).ratio\left(\right)\\ Description(h_{i},h_{j}) & =fuzz.token\_sort\_ratio(des\left[h_{i}\right],des\left[h_{j}\right])/1.0e2\\ Sim(h_{i},h_{j}) & =TE_{ij}\;\cdot \;Location_{ij}\;\cdot \;Category_{ij}\;\cdot \;Description_{ij} \end{array}$$

where:

$h_{i},h_{j}$ = pair of hotels related to the users’ rating;

te        = binarized vector of hotels’ tourist experiences;

cat      = the hotels’ score category string;

des     = the hotels’ services description string;

The CF-CNN model preprocesses the user and hotel identifiers of the rating dataset. Then, the 50-dimensional feature vectors to train and evaluate the algorithm [23] were generated. Initially, the embedment layers transformed the input vectors into matrices and regularized the embeddings using the Gradient Descent (GD) technique [19]. Furthermore, a concatenation layer decreased the dimensionality of the embedding layers. The developed model CF-CNN employed a 1D CNN layer to automatically extract the patterns from the concatenated vector and a Max-Pooling layer to reduce the convolution features map (see Figure 9) [9,19]. Also, a dropout layer to regularize the model during training was added. The FC layers later compressed the extracted features and used a ReLu activation function to produce the predicted rating of the tourist destinations. In contrast to the CF-CNN approach, the embedding matrices-based CF approach (CF-Net) proposed in [23] was implemented. A scalar product between the incrustations (users and hotels matrices) was computed, and, finally, the CF-Net model was trained to apply the GD through a sigmoid function.

The CBF model used the prediction method of KNN [21,22] for estimating the rating of an item based on the average similarity score of the hotels and the ratings registered by a user of the testing dataset.

Finally, the recommendation list was adjusted to 10 items, and the binary vector of TE was added. Also, depending on the predominance of the emotion, the similarity with the TE (cosine similarity in Equation (2)) and the location (Haversine distance in Equation (2)) of the hotels in the top-N list were calculated. The top-N list of tourist recommendations was ordered according to the geographic proximity of the person. Subsequently, the final top-N list of TE recommendations performed better in the proposed algorithms compared to the SVD, SVD++, and normalPredictor algorithms [21].

The source code of the technological components of the ER subsystems and TERS is available in the following public repository: https://github.com/luzsantamariag/terser.

## 5. Experimental Results and Discussion

The datasets were split into a meaningful percentage to train the approaches (80%) and the other percentage to test the performance of the emotional detection and recommendation models.

### 5.1. Emotion Recognition

When evaluating the classification of the imbalanced AV classes, we used k-fold Cross-Validation (CV) to guarantee the presence of all affective states. As defined in Section 4.5, we analyzed the ES dataset with different times between HR instances. Then, we used the ES dataset to predict emotions with shallow ML algorithms and with imbalanced AV classes. Subsequently, we used the parameters of the ES with the best performance to test the ER of the DNN models with balanced AV classes.

#### 5.1.1. Multi-Class Imbalanced Classification

**Figure 10:**
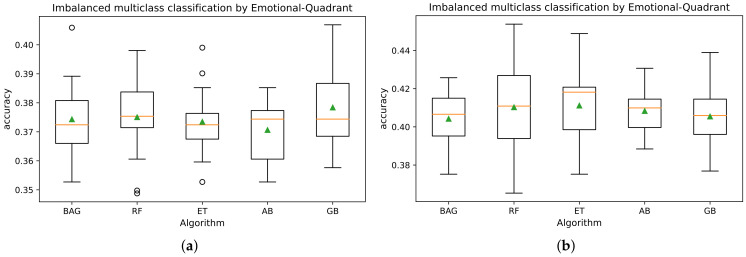
Multiclass classification for ES dataset of 30 HR instances: (**a**) with 60 s between instances; (**b**) with five seconds between instances.

Figure 2 depicts the distribution by emotional quadrant of the physiological dataset and shows an imbalance between the observations of the minority classes (LVHA with 16.3% and LVLA with 10.8%) concerning the majority classes (HVHA with 36.7% and HVLA with 36.2%). Therefore, we used the Scikit-learn library to evaluate the dataset with assembly classification algorithms using stratified 5-fold CV [51]. The dataset was parameterized with ES of 30 HR measurements and different times between instances (60 and five seconds). Figure 10b shows a better performance in the prediction by affective quadrants with less time between instances (five seconds) than the longest time (60 s) (see Figure 10a).

Also, the results of the Random Forest (RF), Gradient Boosting (GB), and Extra Trees (ET) algorithms tend to be slightly better in each case, compared to the Bagging (BAG) and Ada Boost (AB) algorithms that recorded less accuracy in the emotion detection. It should be noted that tests with ES of 20 HR instances were also performed, and the accuracy scores were slightly lower than the tests with 30 HR instances for each ES, as reported in Figure 10. Therefore, the experiments with the DNN models are parameterized with ES of the size of 30 HR instances.

#### 5.1.2. Affective Classification Using DNN Models

The performance of DNN models depends on the volume and quality of the datasets. Therefore, the implementation of heuristic sampling methods compensates for the imbalance in the distribution of affective classes [47,48]. Table 4 shows the results of the DNN models proposed in Section 4.7 for affective detection from the physiological dataset balanced with the K-SMOTE and TL techniques. The three models (1D CNN LSTM, 1D CNN Flatten, and DCNN) used a batch size of 32, with repetitions of 50 epochs and a loss parameter calculated with the categorical cross-entropy function. We configured the Adam optimizer and the learning rate 1e-3 to train the physiological dataset in these models. The accuracy results in the testing were slightly better than shallow ML approaches (see Figure 10).

Combined sampling methods tend to improve accuracy results in both the training and testing of DNN models. Although the AV classes in Table 4 showed an imbalance in positive affective states (HVHA: excited and HVLA: calm) related to negative emotions (LVLA: sad and LVHA: angry), accuracy results performed better with CNN models that used the K-SMOTE and TL data balancing methods. This same trend was confirmed in Table 5, where the emotional class with the lowest number of instances (LVLA: sad) was eliminated. Therefore, the results during training and testing indicate that this dataset with more ES instances may increase the accuracy.

**Figure 11:**
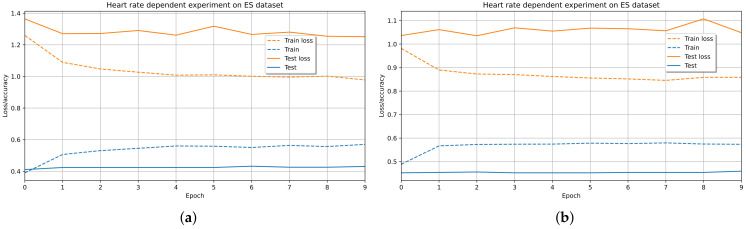
Training and validation of the 1D CNN LSTM model in the Emotional Slices (ES) dataset. The accuracy outcomes correspond to the classification of: (**a**) four emotional quadrants in Table 4; (**b**) three emotional quadrants in Table 5.

The 1D CNN LSTM model showed better performance in detecting AV classes (see Figure 11a,b). However, the efficiency of ER models could be affected by imbalanced spontaneous emotion data and poor measurements of people’s HR. Hence, we defined the dataset evaluation protocol by grouping emotions by VA classes due to the importance of including all affective states during training and testing. Furthermore, we compared the MIC between ES of 30 HR measures with different times between instances and showed that the ES dataset of 5-second interval HR instances performed better. Likely, this ER framework will enhance the accuracy outcomes obtained in a new controlled experiment with more participants to consolidate a more robust dataset.

In this way, we got the prediction of the emotional quadrant. The analysis of the distribution of emotions showed a high rate of participants who registered positive emotions (happy: HAHV and calm: LAHV) instead of negative emotions (LALV: sad and HALV: anger). Therefore, we show that people increasingly seek to improve their TE. Although the imbalance of the emotional dataset limited the prediction results of the ER models, we achieved a better performance of 44% accuracy in the 1D CNN LSTM approach in contrast to the shallow ML algorithms of 41% (see the middle part of Figure 4).

### 5.2. Evaluation of the TERS-ER

The evaluation determined the effectiveness of the approaches proposed in the TERS-ER architecture using the emotional and destinations datasets (see Table 3). The evaluation was carried out with the Mean Absolute Error (MAE) and Root Mean Square Error (RMSE) metrics. These accuracy metrics estimate the average prediction error based on the closeness of the predicted hotel ratings and the actual data (see Equations (3) and (4)). The best performance tends to a zero value, while a result equal to or greater than one indicates a high error rate in the estimation.

The first CBF model was implemented using the Surprise [21] framework. For this reason, the performance tests were compared with the SVD and SVD++ matrix factoring algorithms. The second CF-CNN model compared its performance with the CF-Net algorithm during the training and testing phases. Subsequently, we analyzed the performance results of the proposed models in comparison with the base algorithms.

(3) $$\begin{array}{cc} \mathrm{MAE}= & \frac{\sum_{(i,j)\in TS}\left|r_{ij}-{\hat{r}}_{ij}\right|}{\left|TS\right|} \end{array}$$

(4) $$\begin{array}{cc} \mathrm{RMSE}= & \sqrt{\frac{\sum_{(i,j)\in TS}{\left(r_{ij}-{\hat{r}}_{ij}\right)}^{2}}{\left|TS\right|}} \end{array}$$

where:

TS = represents the number of ratings of all users in the test set;

$r_{ij}$  = depicts the actual rating of a user $u_{i}$ for the hotel’s TE $h_{j}$;

${\hat{r}}_{ij}$ = represents the estimated rating of a user $u_{i}$ for the hotel’s TE $h_{j}$;

#### 5.2.1. Validation of CBF and Model-Based Approaches

**Figure 12:**
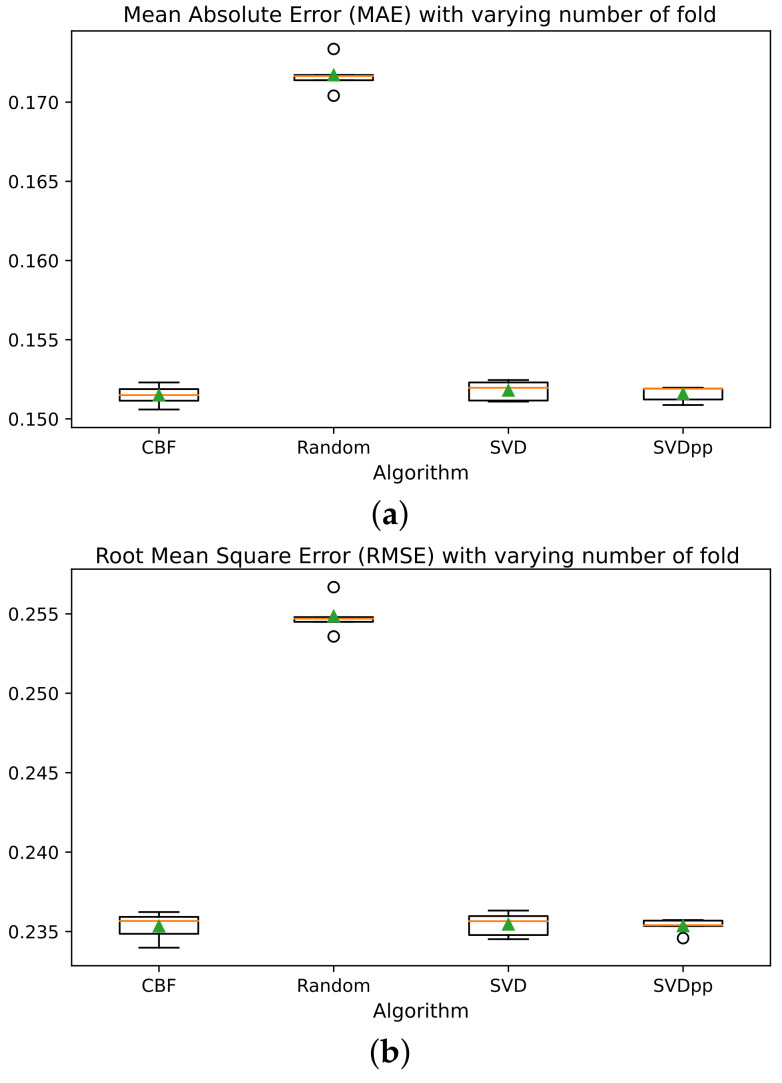
CBF evaluation: (**a**) MAE; (**b**) RMSE.

The results of the tourist datasets training had a positive effect on the performance of the CBF model compared to the algorithms for reducing the dimensionality of latent factors since the CBF algorithm, unlike matrix factorization, correlated the similarity of hotel destinations through the features of TE, location, and description. Moreover, Figure 12 depicts a similar behavior in the five folds of the CV of the algorithms CBF, SVD [58], and the model derived from the latter with the addition of implicit feedback information SVD++ [59]. Further, it shows the distribution of performance measurements and the rising rate according to the hotels’ TE dataset size.

#### 5.2.2. Training and Testing of CF Models

The hotels’ TE datasets were used for evaluating the CF models during the training and testing. The validation parameters of the models were defined by the batch size of 64, a loss function MeanSquaredError (MSE), repetitions of 10 epochs, Adam optimizer, and learning rate of 1 × 10 ^−3^.

**Figure 13:**
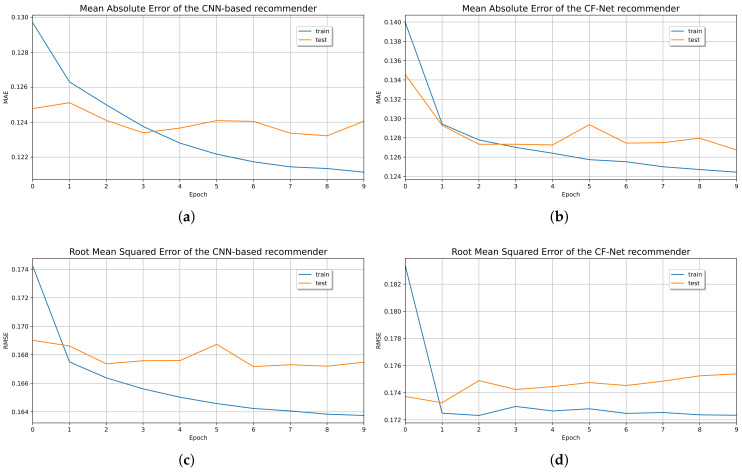
Recommender system evaluation over (**a**) MAE of CF-CNN model; (**b**) MAE of CF-Net model; (**c**) RMSE of CF-CNN model; (**d**) RMSE of CF-Net model.

Figure 13 shows the iterations of the recommendation models during the training and testing with their respective MSE losses. The MAE metric in training and testing shows our CF-CNN model’s better performance than the CF-Net model.

**Figure 14:**
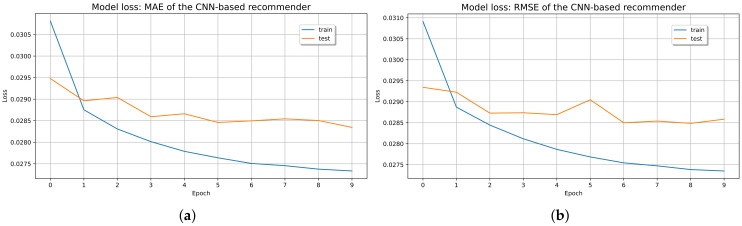
Loss value of CF-CNN (**a**) MAE; (**b**) RMSE.

Therefore, the CF-CNN capacity increased by reducing the regularized overfit by a 0.1 dropout. Furthermore, the loss of the model denotes a positive impact on the training and testing data and is well below 0.1 (see Figure 14).

Likewise, Figure 13 shows the RMSE variation of the epochs of the CF models during the training and testing with their MSE losses. The behavior of the iterations is very similar in both metrics. Also, the performance results of the CF-CNN model are better than those of the CF-Net model.

#### 5.2.3. Comparison of Performance Metrics

**Figure 15:**
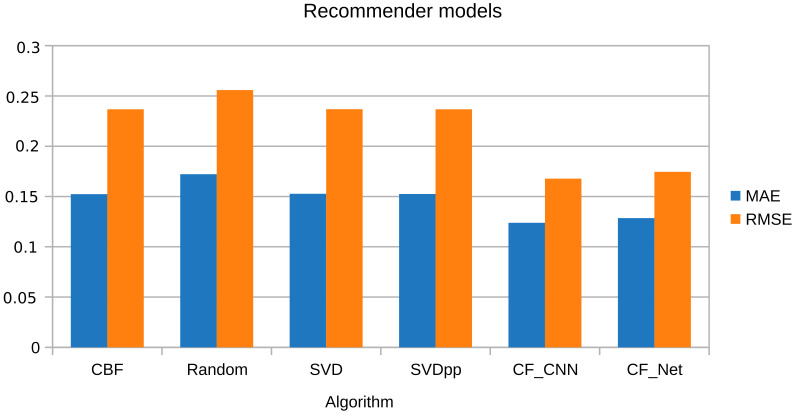
TERS-ER Evaluation.

Unlike the CF-CNN model, the CBF model incorporated similarity metrics between destinations to estimate the rating. Precisely, Figure 15 confirms that the proposed approaches outperform the MAE performance for the predicted rating of the recommendation of tourist destinations concerning the matrix factoring algorithms. However, in the RMSE metric, the model’s performance is better for the CF-CNN approach than the other models.

Furthermore, Table 6 describes the performance of the proposed models and indicates an outstanding improvement in the accuracy of the list of top-N TE. Since the evaluation metrics in both MAE and RMSE were the lowest in the CF-CNN model. In addition, the experimental results with the tourist datasets of the traditional recommendation models had a slightly lower performance than the models based on DNN.

The general outcomes show that the information of the TE, the geographic location, and the attributes of the tourist destinations can affect the performance in the prediction. Finally, the performance results show that the proposed CF-CNN and CBF algorithms perform better in the TERS-ER architecture.

## 6. Conclusions

The proposed architecture is a reference for developing recommendation systems based on users’ emotional states in different contexts. Furthermore, this model allows adding wearable devices with more accuracy physiological sensors [11,13,31,60]. However, when cheap wearable devices become more popular in the market, manufacturers will probably include more accuracy sensors for monitoring biosignals and physical activity [28,29,34,35,36,37]. For this research, we opted for massive and cheap devices that are probably the most used by people in their daily lives. The disadvantage of these devices is the low accuracy of measuring physiological signals that would be very sensitive in medical or specialized applications but are tolerable precisions for tourism. For this reason, an accuracy of 44% in the emotion detection is tolerable to maximize the user experience of these types of devices. Also, it’s important to take into account that this accuracy can be improved with new versions of the wearables used, as with a more robust ES dataset, through another experiment with a more significant number of participants and controlled elicitations, which serves as a cold start for the TERS. It could involve other physiological signals different from HR, such as, for instance, electrodermal activity and temperature.

Regarding the related work represented in Table 2, the use of shallow ML classifiers with an accuracy of around 0.7 can be seen. Our experiment used the 1D CNN-LSTM hybrid classifier with an accuracy result of 0.44. This level of performance is tolerable due to the significant differences in the conditions of the design of the experiments (see Table 7). However, the conditions of this study were planned to meet the requirements of the context, that is, anyone in their daily lives that uses a cheap wearable device. The emotion detection performance of this system is acceptable for recognition, generating an additional contextual factor to a recommender system to improve its accuracy. This contextual factor is emotion, which is a new contribution to recommender systems for the domain of tourism.

Regarding the analysis of RS-related works based on emotions, these works focused mainly on analyzing sentiments of reviews. Their MAE and RMSE results are very close to 1 (see Table 3). On the contrary, in our study, the CF-CNN and CBF classifiers were used, the similarity between users was determined, and the context, preferences, and profile were taken into account. This way, optimal MAE and RMSE results were achieved compared to the other studies (see Section 5.2).

Concerning MIC and following the comparison of results (see Section 5.1.1), it is recommended to parameterize the number of sufficient physiological instances for each ES. In the experiment, better results were obtained with 30 instances of HR for each ES, with a distance between instances of five seconds.

It is necessary to deal with the imbalance of emotion classes in this ER system, which is logical since human behavior predominates in certain emotional types, although the contextual stimulus differs. For instance, a happy person tends to feel more frequent emotions from the happiness quadrant (HVHA). Then in the emotional register, an imbalance of classes is created for the other quadrants. For this reason, combined K-SMOTE and TL techniques were applied for balancing the minority emotional classes. It was also experimented with the elimination of the instances of a minority class, in this case, sadness (LVLA), to improve the performance of the classifier, although the performance improved (see Table 4 and Table 5). This procedure is not recommended because it biases the emotional behavior and this can lead to overfitting of the model.

The main contributions of this research were:

- The TERS-ER model (see Section 4)
- An algorithm that synchronizes the emotional labeling of a physiological time series in an adjustable and sliding window (see Section 4.5).
- An algorithm that creates emotional segments (see Section 4.6) according to the process of an emotion formulated by Norman [14].
- The MEB app (see Section 4.4) replaces the paper recording of the emotional spectrum that was done with SAM.
- An emotional dataset, heart rate recording, and emotion recording were created from the data collection of the Xiaomi Mi Band wearable devices and the MEB app of 18 participants of the experiment (see Section 3).
- Source code for the implementation of the TERS-ER model (see Section 4.8).

Future research would focus on merging multimodal physiological datasets to the TERS-ER architecture to optimize the affective detection of users. The system could incorporate contextual information on the environment and travel routes to increase user satisfaction.

Furthermore, this research is part of the second of five phases of a TE recommendation macro-project. Future areas of research would involve the following:

- Definition of an emotion recognition model from a publicly available emotional dataset. In this case, the AMIGOS dataset was used [16].
- Definition of the model: This corresponds to the results of this paper, where we defined the TERS-ER architecture.
- Consolidation of the ES dataset for the cold start: Replicating the experiment on a larger scale and in a controlled environment to consolidate a large ES dataset with better performance indicators in emotional detection from HR data.
- TERS-ER production: users in the context of their daily life, months or weeks before planning their TE, use low-cost wearable devices (Xiaomi mi band) and the application (TE recommender) on their smartphone to collect in this period the HR data. Subsequently, the HR data collected from the user will be labeled with the emotions according to the ES dataset, and the remaining stages of the model are applied to make the respective recommendation.
- Improvement of the TERS-ER: Defines the ability of the dataset to learn by itself from the new instances generated by the production environment, using advanced ML techniques such as, for instance, reinforcement learning or deep reinforcement learning [19].

## Figures and Tables

**Table 1:** Performance of DCNN and shallow ML algorithms using AMIGOS dataset [16].

Classifier of Emotion Detection	GSR Signals				ECGL Signals			
	Arousal		Valence		Arousal		Valence	
	Accuracy	F1-Score	Accuracy	F1-Score	Accuracy	F1-Score	Accuracy	F1-Score
Naive Bayes [15]		0.54		0.53		0.59		0.57
Nearest Neighbors	0.68	0.64	0.69	0.68	0.69	0.66	0.58	0.57
Linear Discriminant Analysis	0.67	0.61	0.64	0.55	0.72	0.63	0.67	0.65
Linear Support Vector	0.69	0.56	0.68	0.55	0.68	0.6	0.61	0.55
Multi-Layer Perceptron	0.68	0.6	0.64	0.55	0.68	0.59	0.61	0.51
AdaBoost	0.64	0.59	0.66	0.65	0.7	0.66	0.61	0.58
Random Forest	0.58	0.58	0.64	0.64	0.68	0.67	0.59	0.59
DCNN [16]	0.71	0.68	0.75	0.71	0.81	0.76	0.71	0.68

**Table 2:** Emotion detection studies based on physiological data from wearable devices.

Reseach	Wearable		Method			Emotion Detection		
	Technology	Low-Cost	Dataset	Experiment	Participants	Signal	Classifier	Accuracy
[27]	Electrodes	No	DEAP	Controlled	32	GSR and PPG: Covariance matrix	Random Forest	0.72 A and 0.71 V
[30]	Electrodes	No	DEAP	Controlled	32	EEG: Time domain	LERM	0.73 A and 0.74 V
[16]	Electrodes	No	AMIGOS	Controlled	40	GSR and ECG: SCR peak and R-peak	DCNN	A (0.71, 0.81) and V (0.75, 0.71)
[12]	Garmin Vívosmart 3	No	(own dataset created)	Controlled	17	PPG: IBI (Frequency and Time domain)	Bayesian DNN	F1 score: 0.7 V
[13]	Empatica E4	No	(own dataset created)	Controlled	20	PPG: HR	SVM	0.46: HVHA, HVLA, LVHA, LVLA
This study	Xiaomi mi band	Yes	(own dataset created)	Semi controlled	18	PPG: HR	1D CNN-LSTM	0.44: HVHA, HVLA, LVHA, LVLA

**Table 3:** Studies of recommendation systems based on emotions.

Research	Dataset	Algorithms	Similarity	Result
[41]	312,896 Tongcheng reviews and 5722 destinations	UBCF, IBCF, and TF-IDF (scenery, cost, infrastructure, accommodations, traffic, and travel sentiments)	CS	MAE and RMSE: Hybrid CF (0.63, 0.97) and TopicMF (0.76, 1.04)
[42]	TripAdvisor and Yelp: 48,253 POI, 33,576 users, and 738,995 ratings.	Emotion Induced UBCF and Emotion Induced IBCF	CS	Precision: 0.74 UBCF, 0.66 IBCF, and 0.67 Hybrid
[43]	312,896 Tongcheng reviews and 5722 destinations	Syn-ST SVD++ model: sentiment tendency and temporal factors dynamic	PCC	MAE and RMSE: Syn-ST SVD++ (1.04, 0.91)
[44]	TripAdvisor and Yelp: 48,253 POI, 33,576 users, and 738,995 ratings.	HSS (AKNN and SPTW) and AbiPRS (Fuzzy-C-means).	User cluster	Precision and MAE: HSS (0.81, 0.63) and AbiPRS (0.77, 0.73)
This study	OntoTouTra [20]: 1939 TE, 42,202 users, and 530,294 ratings	CF-CNN and CBF	CS	MAE and RMSE: CBF (0.15, 0.23) and CF-CNN (0.12, 0.16)

**Table 4:** ES dataset performance with CNN-based ER models and four-class balancing methods.

Model	Data Balancing Method	Dataset		Train Accuracy		Test Accuracy	
		Labels	HR Slices	Better	Average	Better	Average
DCNN [16]	K-SMOTE	HVHA, HVLA,	1231, 1141,	0.60	0.56	0.46	0.41
	K-SMOTE + TL	LVLA, LVHA	200, 456	0.61	0.57	0.44	0.43
1D CNN, Flatten, and FC	K-SMOTE	HVHA, HVLA,	1231, 1141,	0.65	0.61	0.45	0.41
	K-SMOTE + TL	LVLA, LVHA	200, 456	0.69	0.64	0.46	0.43
1D CNN, LSTM, and FC	K-SMOTE	HVHA, HVLA,	1231, 1141,	0.63	0.58	0.47	0.42
	K-SMOTE + TL	LVLA, LVHA	200, 456	0.67	0.63	0.46	0.44

**Table 5:** ES dataset performance with CNN-based ER models and three-class balancing methods.

Model	Data Balancing Method	Dataset		Train Accuracy		Test Accuracy	
		Labels	HR Slices	Better	Average	Better	Average
DCNN [16]	K-SMOTE	HVHA, HVLA,	1231, 1141,	0.54	0.53	0.48	0.45
	K-SMOTE + TL	LVHA	456	0.58	0.58	0.46	0.46
1D CNN, Flatten, and FC	K-SMOTE	HVHA, HVLA,	1231, 1141,	0.63	0.57	0.50	0.46
	K-SMOTE + TL	LVHA	456	0.67	0.62	0.50	0.47
1D CNN, LSTM, and FC	K-SMOTE	HVHA, HVLA,	1231, 1141,	0.56	0.54	0.50	0.47
	K-SMOTE + TL	LVHA	456	0.56	0.54	0.51	0.47

**Table 6:** Performance statistics with different TERS algorithms.

Algorithm	MAE	RMSE
CBF (This study)	0.152	0.237
Random [21]	0.172	0.256
SVD [21]	0.153	0.237
SVD++ [21]	0.153	0.237
CF-CNN (This study)	0.124	0.168
CF-Net [23]	0.128	0.175

**Table 7:** Differences between emotion recognition studies.

	Our Study	Related Studies
Context	Daily life	Laboratory
Devices	Cheap wearable	Specialized sensors and wearables
Annotators	Self-annotation (MEB app)	Team of annotators (external and internal)
Participants	18	20 (average)
Stimuli	Daily life–spontaneous	Videos and images–controlled
Emotion duration	Variable	Constant (1–2 min)
Emotion annotation	Voluntary	Mandatory
Experiment duration	11 weeks	1 day
Signals	HR (PPG)	PPG, GSR, EEG, ECG (multi-channel)
Signal recording	Sampling (third-party app)	Continous
Domain	Tourist	Various

## Data Availability

Both the source code and the data generated in this study are available at the following link: https://github.com/luzsantamariag/terser.

## Abbreviations

The following abbreviations are used in this manuscript:

A	Arousal
CBF	Content-Basic Filtering
CF	Collaborative Filtering
CS	Cosine Similarity
CNN	Convolutional Neural Network
DCNN	Deep Convolutional Neural Network
DL	Deep Learning
DNN	Deep Neural Network
ECG	Electrocardiogram
EEG	Electroencephalogram
ER	Emotion Recognition
ES	Emotional Slicing
FC	Fully Connected
GSR	Galvanic Skin Response
HR	Heart Rate
HVHA	High Valence High Arousal
HVLA	High Valence Low Arousal
IBI	Inter-Beat-Interval
IoT	Internet of Things
KNN	K-Nearest Neighbor
LSTM	Long Short-Term Memory
LVHA	Low Valence High Arousal
LVLA	Low Valence Low Arousal
MAE	Mean Absolute Error
MEB	MyEmotionBand app
MFB	Master For mi Band
MIC	Multi-class Imbalanced Classification
ML	Machine Learning
NLP	Natural Language Processing
OntoTouTra	Ontology for Tourist Traceability
OTA	Online Travel Agency
POI	Point of Interest
PPG	Photoplethysmogram
RF	Random Forest
RMSE	Root Mean Square Error
RS	Recommender System
SAM	Self-Assessment Manikin
SMOTE	Synthetic Minority Oversampling Technique
SPARQL	SPARQL Protocol and RDF Query Language
SVM	Support Vector Machine
TE	Tourist Experiences
TERS	Tourist Experiences Recommender System
TERS-ER	Tourist Experiences Recommender System based on Emotion Recognition
TL	TomekLinks
V	Valence
